# A novel plastic stent to prevent dislocation after endoscopic ultrasound-guided gallbladder drainage

**DOI:** 10.1055/a-2749-2892

**Published:** 2025-12-11

**Authors:** Kazuki Hama, Hiroyuki Kojima, Takayoshi Tsuchiya, Reina Tanaka, Ryosuke Tonozuka, Shuntaro Mukai, Takao Itoi

**Affiliations:** 1 13112 Department of Gastroenterology and Hepatology, Tokyo Medical University, Tokyo, Japan

Endoscopic ultrasound-guided gallbladder drainage (EUS-GBD) using plastic stents had been originally developed for patients unfit for surgery 1 2 . Lumen-apposing metal stents (LAMSs) are being widely used due to their superior drainage capability and ease of deployment 3 . However, LAMSs are not designed for permanent placement, and subsequent internal fistulization with plastic stents is typically required to maintain long-term drainage 4 . Conventional double-pigtail plastic stents may be insufficient to maintain large fistula tracts, with the risks of spontaneous dislocation and recurrence of cholecystitis. To address this limitation, we developed a novel plastic stent specifically designed to prevent dislocation ( Fig. 1 ; Video 1 ).

**Fig. 1:**
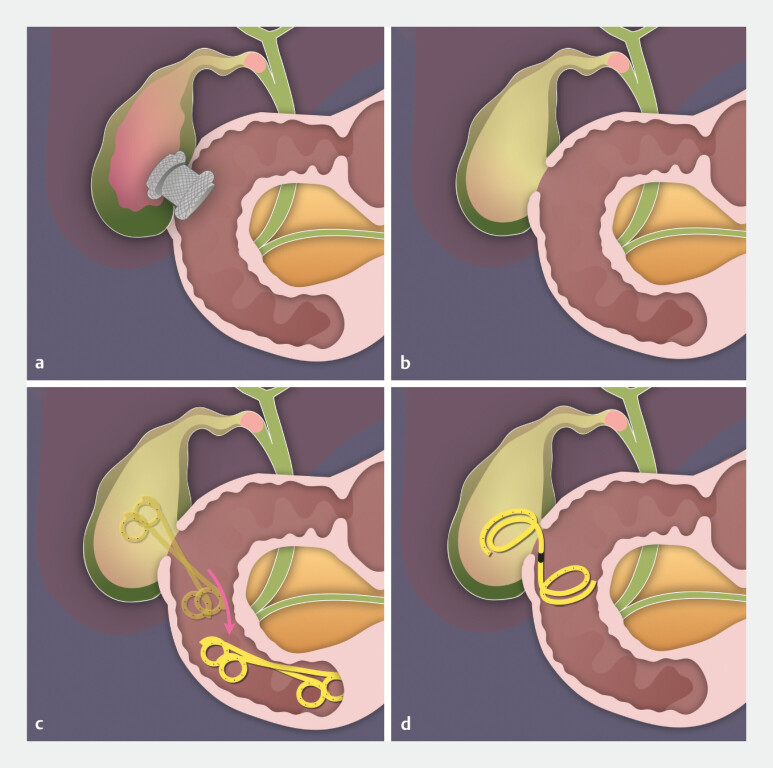
**a** A 10-mm lumen-apposing metal stent (LAMS) was placed. **b** Removal of the LAMS left a large fistula. **c** Conventional double-pigtail plastic stents may be insufficient to maintain large fistula tracts. **d** A newly designed plastic stent prevents dislocation.

A novel plastic stent may serve as a reliable bridging device after LAMS removal in EUS-GBD. EUS-GBD, Endoscopic ultrasound-guided gallbladder drainage; LAMS, lumen-apposing metal stent.Video 1

A 60-year-old man with a history of cerebral infarction presented with right hemiplegia and severe dysphagia. Communication was limited, and percutaneous endoscopic gastrostomy (PEG) was considered. During hospitalization, he developed acute cholecystitis due to gallstones. Because of his poor general condition, surgical cholecystectomy was deemed unfeasible, and the patient was classified as a “never surgery” case.

EUS-GBD was performed using a 10-mm LAMS system (Hot AXIOS; Boston Scientific Co.) ( Fig. 2 ). Drainage was effective, and clinical inflammation subsided rapidly. Three weeks later, the LAMS was removed, and a newly designed plastic stent was placed to maintain the cholecystoenteric fistula ( Fig. 3 ). This novel stent features an anti-migration design and sufficient caliber to maintain fistula patency while minimizing the risk of spontaneous dislocation. After placement, the patient showed no signs of stent dislocation, occlusion, or recurrent infection. No re-intervention was necessary, and clinical progress remains favorable ( Fig. 4 ).

**Fig. 2:**
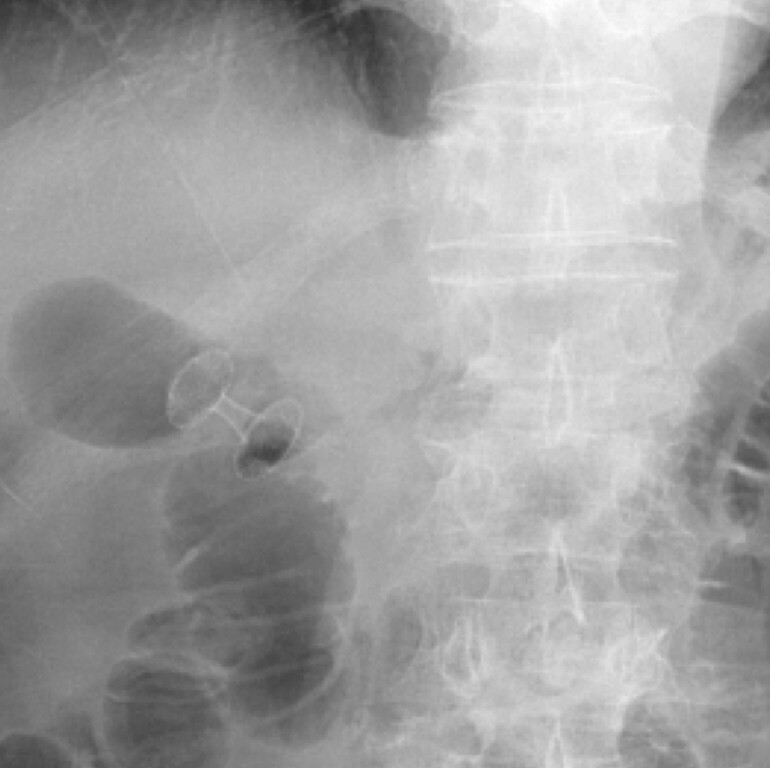
A 10-mm lumen-apposing metal stent (LAMS) was placed.

**Fig. 3:**
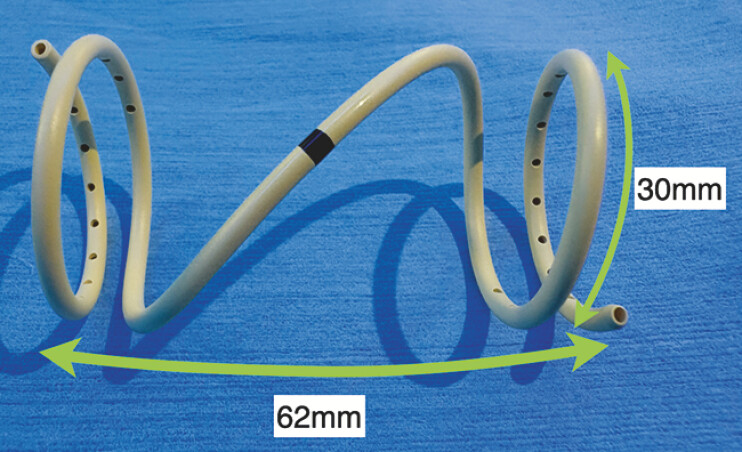
The novel plastic stent was 62 mm long, and the pigtail portion had a maximum diameter of 30 mm.

**Fig. 4:**
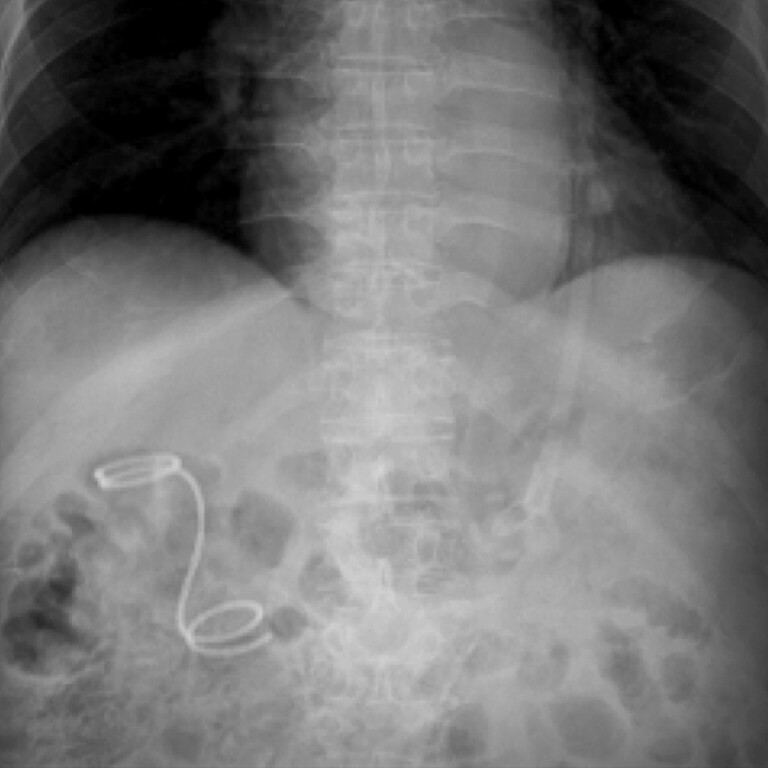
After placement, the patient showed no signs of stent dislocation, occlusion, or recurrent infection.

This case highlights the potential clinical utility of the new plastic stent as a bridging device after LAMS removal in EUS-GBD. It may offer a safer and more reliable long-term internal drainage option for patients who are not surgical candidates.

Endoscopy_UCTN_Code_TTT_1AS_2AH

## Notes

Endoscopy E-Videos https://eref.thieme.de/e-videos
*E-Videos* is an open access online section of the journal *Endoscopy* , reporting on interesting cases and new techniques in gastroenterological endoscopy. All papers include a high-quality video and are published with a Creative Commons CC-BY license. Endoscopy E-Videos qualify for HINARI discounts and waivers and eligibility is automatically checked during the submission process. We grant 100% waivers to articles whose corresponding authors are based in Group A countries and 50% waivers to those who are based in Group B countries as classified by Research4Life (see: https://www.research4life.org/access/eligibility/ ). This section has its own submission website at https://mc.manuscriptcentral.com/e-videos .
